# Cholera as It Prevailed in New Orleans

**Published:** 1849-03

**Authors:** E. D. Fenner


### SELECTIONS

FROM

### AMERICAN AND FOREIGN JOURNALS.

---

*Cholera as it prevailed in New Orleans.* By E. D. Fenner, M.D.—Dr. Fenner has an interesting letter on the late epidemic in the daily Picayune of the 28th January, from which we make the following extracts. The communication was prepared for the New Orleans Medical and Surgical Journal, but being too late for the February number, the author concluded to send it forth through the present medium. Dr. Fenner remarks that stomach and bowel complaints were prevalent in the city before the Swanton, charged with the introduction of cholera, arrived. He says—

On the 5th of December, (the Swanton arrived on the 11th), I attended a gentleman on Customhouse street, who labored under vomiting, pain and spasms in the bowels, and prostration to such a degree that, if epidemic cholera had been supposed to be here, no person would have hesitated to pronounce him a case. He had no rice water evacutions, but his bowels were rather costive, and he vomited bile; but many such cases have been seen since the epidemic was declared. He recovered after two or three days' illness, and has not been again sick.

Some days previous to this, three or four negroes were attacked with cholera morbus on the same night and at the same house, in Gravier street. They were promptly treated, and all soon recovered. Similar cases were observed in the practice of a number of physicians in different parts of the city, all going to show, as it appears to me, that the epidemic influence of cholera was gradually being matured and developed in our midst.

*Chloroform successfully used.*—He says: On the evening of the same day (13th December) that the second case was taken to the Charity Hospital, a man who has resided here for many years, who does business not far from the St. Charles Hotel, came into my office with strong symptoms of cholera. He had not been near the ship Swanton, nor seen any of the passengers. I prescribed for him, and on visiting him at his room, half an hour afterwards, I found him extremely ill, with severe pain in the bowels, copious watery purging, skin bathed in cold sweat, great thirst, and general prostration. His condition was so alarming and he derived so little relief from large and repeated doses of opium, calomel, camphor, and capsicum, assisted by sinapisms, stimulating frictions, etc., that I determined to resort to the inhalation of chloroform. By this means he was made perfectly easy in about two minutes, and remained so until the other medicines he had taken had time to act. He got through the night pretty well, and recovered in a few days from a dangerous illness.

*Death of a medical man from his obstinacy in refusing to take medicine.*—On the 16th of December, remarks Dr. Fenner, the disease was rapidly increasing, when he was called to the following case:

Dr. J. B. Morgan, of Jackson, Miss., was attacked the night previous, without having committed any other indiscretion than eating some fish and oysters at dinner. When I arrived at his room, I met Dr. Farrell, who had seen him before and had good reason to be provoked at the difficulty he found in convincing Dr. Morgan of the danger he was in and the importance of prompt and vigorous treatment. Dr. Morgan had already passed about thirty liquid evacuations, then had cramps in his legs, and in fact was on the verge of collapse. Dr. M. being an old friend and neighbor of mine, I joined my entreaties to the arguments of Dr. Farrell, and we did all we could to convince him of the importance of vigorous treatment, but all to no purpose. He insisted that he was not dangerously ill—that he had been similarly affected many a time before, and that if he were not disturbed he would soon be well. The sequel verified our worst apprehensions. He was incorragibly obstinate, dallied too long with a dangerous disease, and was lost.

*Relation of the weather to the epidemic.*—From the 16th to the 22d the weather was oppressively warm, the thermometer rising as high as 84°. From the 22d it was cool, wet, and gloomy, till the night of the 30th, when there fell a white frost. On the morning of the 1st of January there was another white frost, and from that time the disease declined steadily.

*Character of the epidemic.*—As to the character of the epidemic, I think I may safely say that it has not been very malignant. In most instances the attack was insidious and mild—generally commencing with a looseness of the bowels, attended with more or less griping, and often accompanied with nausea and vomiting. The latter symptoms almost invariably attended those patients who had committed imprudence in eating. Without descending into minutiæ, I may say that the disease almost invariably commenced with some unusual disturbance of the digestive organs. When this disturbance commanded the attention it deserved, it was generally most easily remedied by the simplest means; but if neglected, it seldom failed to lead on to the most disastrous consequences. This, then, is the *curable stage* of cholera, and almost the only stage in which it can be cured; for if it be permitted to run on till the patient becomes cold and pulseless, ninety-nine in a hundred will inevitably die. By powerful means, reaction may often be established; but the danger is not yet passed—a great majority still die of the consecutive fever. Say what you will about creating *panic* and spreading alarm among the people, I feel no hesitation in asserting that when epidemic cholera is prevailing, every person who has any unusual diarrhea, had better believe he is *a case* and act accordingly. If this simple rule were universally adopted, cholera would soon be rendered comparatively harmless. Thus, according to Dr. Watson, one of the ablest English authors, the disease was arrested in London by the establishment of "*Diarrhea Dispensaries*," where the poor were supplied gratuitously with proper remedies. Thousands applied, who would otherwise have waited to *get ill* before going to a hospital to be treated. Who can deny that it would be well to *frighten* the people, if need be, into this degree of precaution? Without it you may rest assured there is no safety.

I deem it unnecessary to enter into a minute detail of the symptoms that characterize cholera, as they are familiar to most persons, whether belonging to the medical profession or not. It may not be amiss, however, to mention a few of the most remarkable ones that attended the late epidemic. Many bad cases were marked by an obstinate vomiting of bile, which continued until death. The vomiting would continue for days, and incredible quantities would be thrown up. In these cases the diarrhea was generally moderate and sometimes absent. The worst cases were those in which the rice water discharges were profuse, as well from the stomach as the bowels. In these, there was no appearance of bile whatever.

A few cases were seen at the Charity Hospital which terminated in black vomit.

When collapse supervened early in the attack, before the system was too much exhausted by copious rice-water evacuations, it was less difficult to bring about reaction, and there was better hope of ultimate success. So far as I know, the only cases of recovery which took place after decided collapse, were of this kind.

Where reaction from a state of collapse was slow and difficult, a sort of typhus fever supervened, which lasted for some days and generally terminated fatally with affection of the brain. The intellect was generally clear and undisturbed to the last, excepting those who died of the consecutive fever. It is marvellous and astounding to witness the mental clearness and composure of some persons dying with cholera! Whilst the attendant relatives and friends are agonized with grief at the sudden and awful calamity, the poor victim is often seen supernaturally calm and uttering words of consolation with the expiring breath.

*Manageable in the forming stage.—Fatality from quack medicines.*—The epidemic influence appeared to be felt by almost every person in the city, whether native or foreigners, acclimated or unacclimated. Thousands complained of an extraordinary unesiness in the stomach and bowels, but in a vast majority of instances it was esily relieved, and but few bad cases occurred amongst those who were prudent, and paid proper attention to the premonitory symptoms. The lower classes of people have evidently suffered most, which may be attributed to ignorance or neglect. The mortality at the Charity Hospital has been very great; yet no one can be surprised at it who visited that institution during the epidemic and witnessed the condition in which the patients were when admitted. Cholera is an insidious disease that generally steals upon its victims, seldom declaring itself openly until it has them completely within its fatal grasp. I have not a doubt that seven-tenths of the people who have recently perished of it in this city might have been saved, if they had procured proper medical aid at the onset of the disease. I presume there is not a physician in the city who has not been called to persons reduced to the most dangerous condition by relying too implicitly and too long upon some of the various "specifics" advertised in our newspapers and lauded by the editors.

*Treatment.*—The treatment of cholera admits of much variation. Educated physicians everywhere concur in the indications to be fulfilled or what is wanted to be done; but amidst the multiplicity of remedies at their command, of course each one resorts to such as he thinks are best adapted to the circumstances of the case. Doubtless the same object may be accomplished by a variety of means, if it can be accomplished at all; and in our choice of remedies we must be guided by observation and experience. In the treatment of what are called the premonitory symptoms, the first indication is —to check the diarrhea as soon as possible, keeping an

6 *

eye at the same time to the important secretions of the liver, kidneys, and skin. For this purpose, physicians very generally resort to opium and its preparations, combined with stimulants and aromatics—or opium with some mercurial, or with quinine. According to the urgency of the symptoms a good prescription may be made of laudanum or paregoric and brandy—or laudanum, spirits of camphor, and tincture of assafetida—or laudanum, essence of peppermint, and compound spirits of lavender—or calomel, opium, and capsicum or camphor—or equal parts of paregoric and tincture of catcehu. The following is a recipe which I found to answer very well, viz:

℞. Sulph. Quinine, ℈i.

Tinct. Opii, ℈iss.

Tinct. Capsici Comp., ʒiii.

Mucilag. Acaicæ with Aqua Cinnamon, f. ℥iv.

M.

Give a tablespoonful and repeat after every two loose stools.

Although opium is so often found in the prescriptions above, I should mention that some physicians disapprove of it and seldom prescribe it in cholera, except by enema. These gentlemen place their chief reliance upon calomel, combined with camphor, capsicum and the like. As to the sulphate of quinine, it appears to be serviceable in almost every kind of disease that occurs in this region. It possesses remarkable intrinsic virtues, and may be used as an adjuvant to many other remedies.

In the treatment of the violent or acute stage of cholera, when there is vomiting, purging, and cramps, we resort to anodynes, antispasmodics, stimulants, calomel, etc., internally, aided by sinapisms, stimulating frictions, etc., externally.

The treatment of the stage of *collapse* is altogether desperate. As before stated, a vast majority of patients die after getting into this condition; and it is no wonder, for the system is then completely drained of its vital fluid. An excessive hemorrhage from a divided artery would not produce a greater prostration than is brought about by the copious serous evacutions of cholera; for all the fluid discharged is abstracted from the blood.

Various remedies are used in this stage, such as sinapisms, blisters, the hot and cold bath, the hot air bath, calomel, carbonate of ammonia, etc., etc. The most remarkable recovery from collapse that I witnessed was effected by very large doses of calomel, washed down with tablespoon doses of laudanum, aided by sinapisms and frictions with spirits of turpentine and sweet oil. This was a lady who was rescued from the very jaws of death. She was afterwards pretty badly salivated, but recovered without serious injury.

Two other physicians attended her with me. In the case of Dr. Morgan, mentioned before, I witnessed the astonishing power of cold water in bringing about reaction from a hopeless state of collapse. Warmth was restored, the pulse returned at the wrist, and life was prolonged two or three days; but still it failed, for the injury sustained was irreparable. The cold bath was administered in this instance at the suggestion of my friends, Dr. Richardson, of Vicksburg, and Dr. Gustine late of Natchez, who said they had derived great benefit from it in the cholera of 1833. Being favorably impressed with the power which the remedy displayed in the case of Dr. M., I resorted to it in two other cases of collapse in private practice; it produced reaction, but they both died for want of vital power to sustain it.

*Mortality of the late epidemic compared with that of* 1832.—The mortality from cholera at its late visitation, compares most favorably with that of 1832, when it first scourged our city. The number of deaths by cholera from the 12th December, 1848, to the 20th January, 1849, as appears from the reports of the Board of Health, amounts to near 1,400, five hundred and ninety-six of which occurred at the Charity Hospital. We learn from an interesting Memoir on the Cholera of 1832, addressed to the Academy of Medicine of Paris, by Dr. M. Halphen, a French practitioner of this city at that time, that the disease made its appearance about the 25th of October, in the midst of an epidemic of yellow fever; that in a few days it raged severely, and that in the short space of twenty days it killed about 6,000 people. Dr. Halphen says, that the mortality amounted on some days as high as 500 a day. He estimates the full population of the city then at 50,000, and as cholera broke out during the prevalence of yellow fever, ere yet the absent citizens had returned, and before the customary visiters dared to come in, he does not think the population at that time exceeded 35,000 thus showing the frightful loss of about *one-sixth of the people in twenty days.*—When we read over these sad details, we may well congratulate ourselves upon our happy deliveranc from the late pestilence. True, we have lost about 1,400 people, amongst them a few valuable citizens; but what would have been our fate if so malignant a disease as that of 1832 had broken out in December last, when all our own people were at home, and the city was full of strangers? In 1832, *the living could not afford decent burial to the dead!*

Dr. Halphen states that on some days upwards of one hundred corpses were accumulated at the cemeteries, waiting for interment. Large trenches were dug, into which cart loads of uncoffined bodies were heaped, indiscriminately; and in the dead of night, a great number of bodies, with bricks and stones tied to the feet, were stealthily thrown into the river. The same ratio of mortality at the present time would demand about 20,000 victims. Let us turn from the appalling calculation, and thank God that we have been so mercifully spared.

As in 1832, the epidemic has declined to a stage of comparative security, but the disease has not entirely disappeared. There is as little cholera in New Orleans at the present time, in proportion to the population, as in any other part of the Lower Mississippi Valley.—Whether the epidemic will be rekindled, at the approach of the ensuing summer, remains to be seen. If the miserable condition of the city, as regards cleanliness, will have any influence upon the event, we may certainly expect it. New Orleans must ever continue to be a prey to the most fatal diseases that prevail, until something efficient is done to improve its sanitary condition.

*Spread of the disease to other parts.*—We learn that cholera is spreading among the plantations along the river, and also in the interior of Louisiana. To some of these the infection appeared to be directly carried; at others it began without any communication with an infected district.

